# Correlation of RANK and RANKL with mammographic density in primary breast cancer patients

**DOI:** 10.1007/s00404-024-07495-1

**Published:** 2024-06-05

**Authors:** Marius Wunderle, Felix Heindl, Annika S. Behrens, Lothar Häberle, Carolin C. Hack, Katharina Heusinger, Hanna Huebner, Paul Gass, Matthias Ruebner, Rüdiger Schulz-Wendtland, Ramona Erber, Arndt Hartmann, Matthias W. Beckmann, William C. Dougall, Michael F. Press, Peter A. Fasching, Julius Emons

**Affiliations:** 1 Department of Gynecology and Obstetrics, Comprehensive Cancer Center Erlangen-EMN (CCC ER-EMN), Erlangen University Hospital, Friedrich-Alexander-Universität Erlangen-Nürnberg (FAU), 91054 Erlangen, Germany; 2 Bavarian Cancer Research Center (BZKF), Erlangen, Germany; 3 Biostatistics Unit, Department of Gynecology and Obstetrics, Comprehensive Cancer Center Erlangen-EMN (CCC ER-EMN), Erlangen University Hospital, Friedrich-Alexander-Universität Erlangen-Nürnberg (FAU), 91054 Erlangen, Germany; 4 Institute of Diagnostic Radiology, Comprehensive Cancer Center Erlangen-EMN (CCC ER-EMN), Erlangen University Hospital, Friedrich-Alexander-Universität Erlangen-Nürnberg (FAU), 91054 Erlangen, Germany; 5 Institute of Pathology, Comprehensive Cancer Center Erlangen-EMN (CCC ER-EMN), Erlangen University Hospital, Friedrich-Alexander-Universität Erlangen-Nürnberg (FAU), 91054 Erlangen, Germany; 6 Immunology in Cancer and Infection Laboratory, QIMR Berghofer Medical Research Institute, Brisbane, QLD 4702 Australia; 7 Hematology and Oncology Research, Amgen, Inc, Seattle, WA 98119 USA; 8 Department of Pathology, Norris Comprehensive Cancer Center, Keck School of Medicine, University of Southern California, Los Angeles, CA 90033 USA

**Keywords:** Breast cancer, Mammographic density, RANK expression, RANKL expression, Immunohistochemistry

## Abstract

**Purpose:**

The receptor activator of nuclear factor kappa B (RANK) and its ligand (RANKL) have been shown to promote proliferation of the breast and breast carcinogenesis. The objective of this analysis was to investigate whether tumor-specific RANK and RANKL expression in patients with primary breast cancer is associated with high percentage mammographic density (PMD), which is a known breast cancer risk factor.

**Methods:**

Immunohistochemical staining of RANK and RANKL was performed in tissue microarrays (TMAs) from primary breast cancer samples of the Bavarian Breast Cancer Cases and Controls (BBCC) study. For RANK and RANKL expression, histochemical scores (H scores) with a cut-off value of > 0 vs 0 were established. PMD was measured in the contralateral, non-diseased breast. Linear regression models with PMD as outcome were calculated using common predictors of PMD (age at breast cancer diagnosis, body mass index (BMI) and parity) and RANK and RANKL H scores. Additionally, Spearman rank correlations (ρ) between PMD and RANK and RANKL H score were performed.

**Results:**

In the final cohort of 412 patients, breast cancer-specific RANK and RANKL expression was not associated with PMD (*P* = 0.68). There was no correlation between PMD and RANK H score (Spearman’s ρ = 0.01, *P* = 0.87) or RANKL H score (Spearman’s ρ = 0.04, *P* = 0.41). RANK expression was highest in triple-negative tumors, followed by HER2-positive, luminal B-like and luminal A-like tumors, while no subtype-specific expression of RANKL was found.

**Conclusion:**

Results do not provide evidence for an association of RANK and RANKL expression in primary breast cancer with PMD.

## What does this study add to the clinical work?

**Table:** 

The inhibition of the receptor activator of nuclear factor kappa B ligand (RANKL) pathway with denosumab is currently being tested in clinical trials for the primary prevention of breast cancer in women with high breast cancer risk. This study investigated whether the expression of RANK and RANKL in the tumor tissue of patients with primary breast cancer correlates with the well-known breast cancer risk factor mammographic density, and did not find an association.	

## Introduction

High mammographic density (MD) has been confirmed to modify breast cancer risk depending on the percentage of MD (PMD) with a two–sixfold increased risk [1, 2]. Besides from familial and genetic factors [3, 4], higher PMD has been linked with the cumulative exposure to growth factors and hormones. This includes a great lifetime number of menstrual cycles by early menarche and late menopause, which is an indicator for cumulative exposure to luteal phase progesterone levels, a low number of parities and life births, adipose body mass index (BMI), combined estrogen-plus-progestin hormone replacement therapy, elevated levels of prolactin, and other factors [1, 3, 5, 6].

PMD reflects the proportion of dense breast tissue comprising epithelial cells, fibroblasts, and connective tissue on a mammogram, whereas adipose tissue is the main component of non-dense breast tissue. Although it has been proposed that stromal architecture and composition of the breast influence epithelial biology and play an initial role in breast carcinogenesis, the molecular mechanisms between PMD and increased breast cancer risk are still not well understood [2, 3].

The receptor activator of nuclear factor kappa B (RANK) and its ligand (RANKL) as well as osteoprotegerin (OPG), functioning as an antagonistic, soluble decoy receptor for RANKL, are expressed by various tissues and cell lines. Besides its role in bone metabolism and osseous metastasis, RANK/RANKL/OPG signaling is also involved in physiological and pathological processes of immune response and proliferation of different tissues including the mammary gland [7–9].

It has been demonstrated that progesterone and prolactin increase the expression of RANKL in the breast and interact with the RANK pathway, inducing lobulo-alveolar differentiation, proliferation, and expansion of mammary epithelial cells. Inhibition of progesterone, RANK or RANKL resulted in less mammary cell proliferation, carcinogenesis, and metastasis in mouse models [7, 9–11]. This has been shown especially in models of *BRCA1* mutated breast cancer [7, 9, 12, 13].

The monoclonal antibody against RANKL denosumab has proven efficacy in the prevention and treatment of osteoporosis and bone metastases in breast cancer as well as in other types of cancer [14, 15]. In addition, trials with female *BRCA* mutation carriers are investigating the effect of denosumab on proliferation of the breast epithelium (*BRCA-D, ACTRN12614000694617* [16]) and as a chemopreventive drug against breast cancer (*BRCA-P, NCT04711109* [17]).

Because of its association with breast proliferation and mammary tumor development, it has been hypothesized that RANK, RANKL, and OPG expression is linked with PMD. This has been investigated by few studies for serum or plasma expression [18–20], or expression in healthy breast tissue [21], but not for breast cancer-specific expression so far. The aim of the present study was thus to assess the correlation of RANK and RANKL expression in primary breast cancer samples with PMD of the contralateral, healthy breast.

## Patients and methods

### Patients

**Fig. 1:**
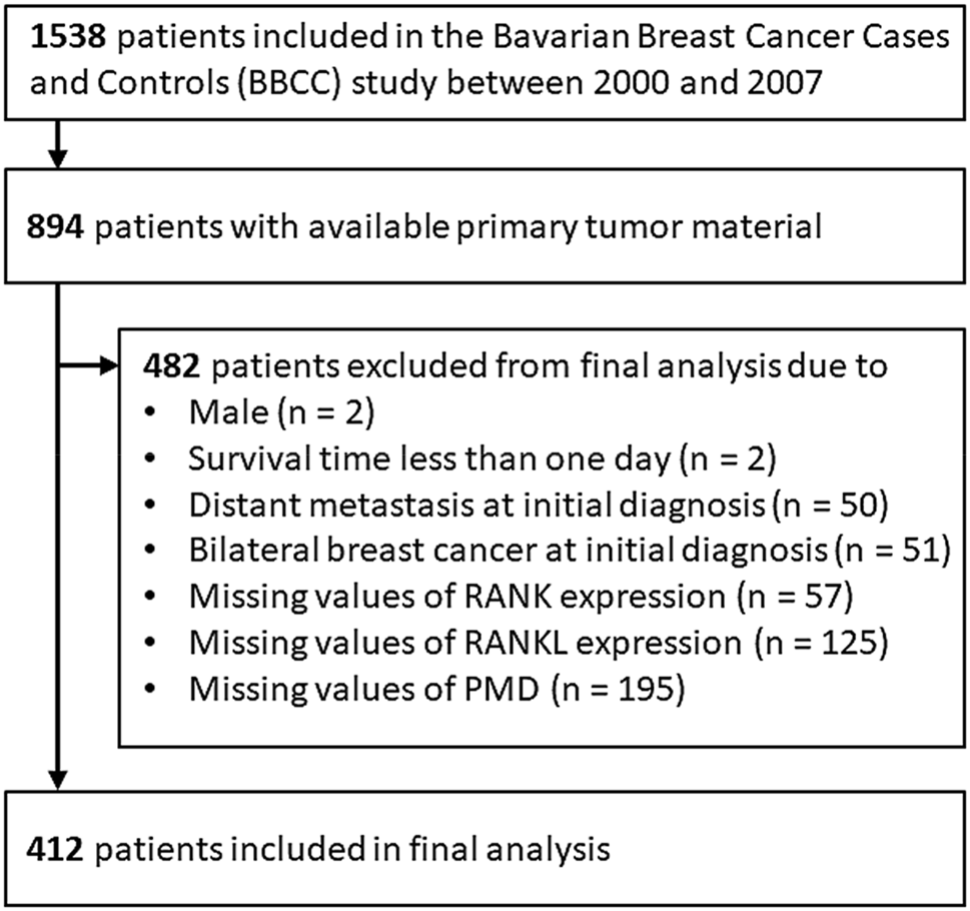
Flowchart of patient selection

The Bavarian Breast Cancer Cases and Controls (BBCC) study is a case–control study investigating molecular and epidemiological breast cancer risk factors as well as prognostic and predictive factors including PMD. Between 2000 and 2007, 1538 patients were included who were at least 18 years old and had a diagnosis of invasive breast cancer. Tissue microarrays (TMAs) were constructed from 894 patients. After exclusion of datasets with ineligible characteristics or missing information, the final study population comprised 412 female patients with unilateral invasive breast cancer. The detailed selection process is provided in Fig. 1.

### Histopathological, epidemiological and follow-up data

Comprehensive data on tumor and patient characteristics as well as follow-up data for a minimum of 10 years after initial diagnosis were documented conforming to the requirements of the German Cancer Society (Deutsche Krebsgesellschaft) and the German Society for Breast Diseases (Deutsche Gesellschaft für Senologie) as part of the certification process [22]. Breast cancer subtypes were defined as previously described [23]. Shortly, HER2 receptor-negative tumors which showed either estrogen receptor (ER) or progesterone receptor (PR) expression (≥ 10%) were classified as luminal A-like for Ki-67 < 15% and grading of 1 or 2 or luminal B-like for Ki-67 ≥ 15% and grading of 2 or 3. HER2 receptor-positive breast cancer was stated in patients with HER2 staining of 3 + as assessed by immunohistochemistry or HER2 gene amplification. Patients with HER2-negative and hormone receptor (HR)-negative or weakly positive (< 10%) breast cancer were considered as triple-negative (TNBC) [23].

### Assessment of PMD

For the assessment of PMD, mammograms were eligible if they were taken 1 year before or 3 months after breast cancer diagnosis. PMD was measured on the contralateral breast, which was not affected by breast cancer in cranio-caudal (CC) projection. In this work, full-field digital mammograms and film-based mammograms were examined. Analog film-based mammograms were digitized by a CadPro Advantage® film digitizer (VIDAR Systems Corporation, Herndon, Virginia, USA). Breast area measurements and quantitative computer-based threshold density assessments were performed by two individual, experienced readers with special training in the applied method. Mammograms were analyzed in an independent and arbitrary order, and the readers were unaware of any previous findings. Finally, the mean PMD of the two readers was used for analysis. The MD proportion was evaluated using the Madena software program, version 3.26 (Eye Physics, LLC, Los Alamitos, California, USA). This method has been validated and described before [24], and we used it in several previous works [1, 6, 25–28].

### Assessment of RANK and RANKL expression

Tumor specimens were formalin-fixed and paraffin-embedded (FFPE). In a first step, an experienced pathologist marked the tumor areas of interest on a hematoxylin–eosin-stained slide. For the construction of TMAs, cylindric tissue core biopsies (0.8 mm per dot) from multiple sample donor blocks were re-embedded in a second step into a single microarray block at predefined coordinates. Staining of the TMA was performed with anti-human RANK (N-1H8; Amgen, Thousand Oaks, California, USA) or RANKL (M366; Amgen, Thousand Oaks, California, USA) mouse monoclonal antibodies or isotype-matched control mouse IgG, as previously described [29, 30]. For each primary tumor, RANK and RANKL expression was scored according to the semiquantitative histochemical score (H score) [31]. Experienced pathologists conducted the immunohistochemical interpretation blinded to any sample identification. The percentage of RANK and RANKL-positive tumor cells was multiplied by staining intensity, respectively: 0, negative; 1 + , weak; 2 + , moderate; and 3 + , strong. The sum of all calculated tumor cell percentage/intensity product for every TMA dot was defined as H score, ranging from 0–300. As a result, 300 represents 100% of tumor cells having a strong staining intensity.

### Statistical analysis

The primary objective of this analysis was to investigate the association between RANK and RANKL tumor expression, quantified as H score, and PMD. For that purpose, we calculated linear regression models with PMD as outcome. The square root of PMD was used to gain normally distributed residuals of the models. First, a basic model with the following predictors was set up: age at diagnosis (continuous), BMI (continuous), and parity (number of children born, categorical: 0, 1, 2, and ≥ 3). Afterwards, RANK (> 0 vs 0) and RANKL (> 0 vs 0) was added to the basic model to obtain a full model. Due to large proportions of zeroes in RANK and RANKL H scores, those variables were dichotomized in either negative or positive to perform further analyses.

The basic and the full model were compared using the F test. A significant result means that RANK or RANKL H score is associated with PMD. As sensitivity analysis we calculated Spearman rank correlations (ρ) between PMD and RANK or RANKL H score and tested their significance.

Subjects with missing values in RANK and RANKL H score and PMD were excluded from analysis. Missing values in other predictors were imputed as described in Salmen et al. [32]. The 15 (3.6%) values for BMI were substituted by the median of non-missing data. For the imputation of the 25 (6.1%) values in parity, we calculated a multinomial logistic regression model with the predictors age, BMI, and PMD.

All of the tests were two-sided, and *P* < 0.05 was regarded as statistically significant. Calculations were carried out using the R system for statistical computing (version 3.4.0; R Development Core Team, Vienna, Austria, 2017).

## Results

### Patient and tumor characteristics

Overall, 412 female patients with primary breast cancer were included in the final analysis. Mean age at breast cancer diagnosis was 58.6 (standard deviation, SD 12.7) years, and median BMI was 25.2 (interquartile range, IQR 22.5–28.6) kg/m^2^. A majority of patients gave birth to two children (40.5%), while a minority was nulliparous (13.6%). Most patients had a pathological tumor size of T1 (*n* = 198, 48.1%) or T2 (*n* = 166, 40.3%), had no lymph node involvement (*n* = 219, 53.2%), and had either luminal A-like (*n* = 133, 32.3%) or luminal B-like (*n* = 154, 37.4%) tumors (Table 1).

**Table 1:** Patient and tumor characteristics

**Characteristic**		
Age at diagnosis (years)	Mean (SD)	58.6 (12.7)
BMI at diagnosis	Median (IQR)	25.2 (22.5–28.6)
Parity (children born)	0	56 (13.6%)
	1	125 (30.3%)
	2	167 (40.5%)
	≥ 3	64 (15.5%)
PMD contralateral	Median (IQR)	0.37 (0.24–0.53)
RANK H score	0	269 (65.3%)
	> 0	143 (34.7%)
RANKL H score	0	369 (89.6%)
	> 0	43 (10.4%)
Tumor stage*	T1	198 (48.1%)
	T2	166 (40.3%)
	T3	21 (5.1%)
	T4	27 (6.6%)
Lymph node status*	N negative	219 (53.2%)
	N positive	193 (46.8%)
Molecular subtype	Luminal A-like	133 (32.3%)
	Luminal B-like	154 (37.4%)
	HER2-positive	58 (14.1%)
	Triple-negative	67 (16.3%)

Mean (standard deviation, SD) or median (interquartile range, IQR), where appropriate, are shown for continuous characteristics and frequency (percentage) for categorical characteristics. *In patients undergoing neoadjuvant chemotherapy, the initial clinical tumor and/or lymph node stage was used if the tumor was pathologically downstaged under neoadjuvant chemotherapy. *BMI* body mass index, *PMD* percent mammographic density

### RANK and RANKL expression and correlation with PMD

**Fig. 2:**
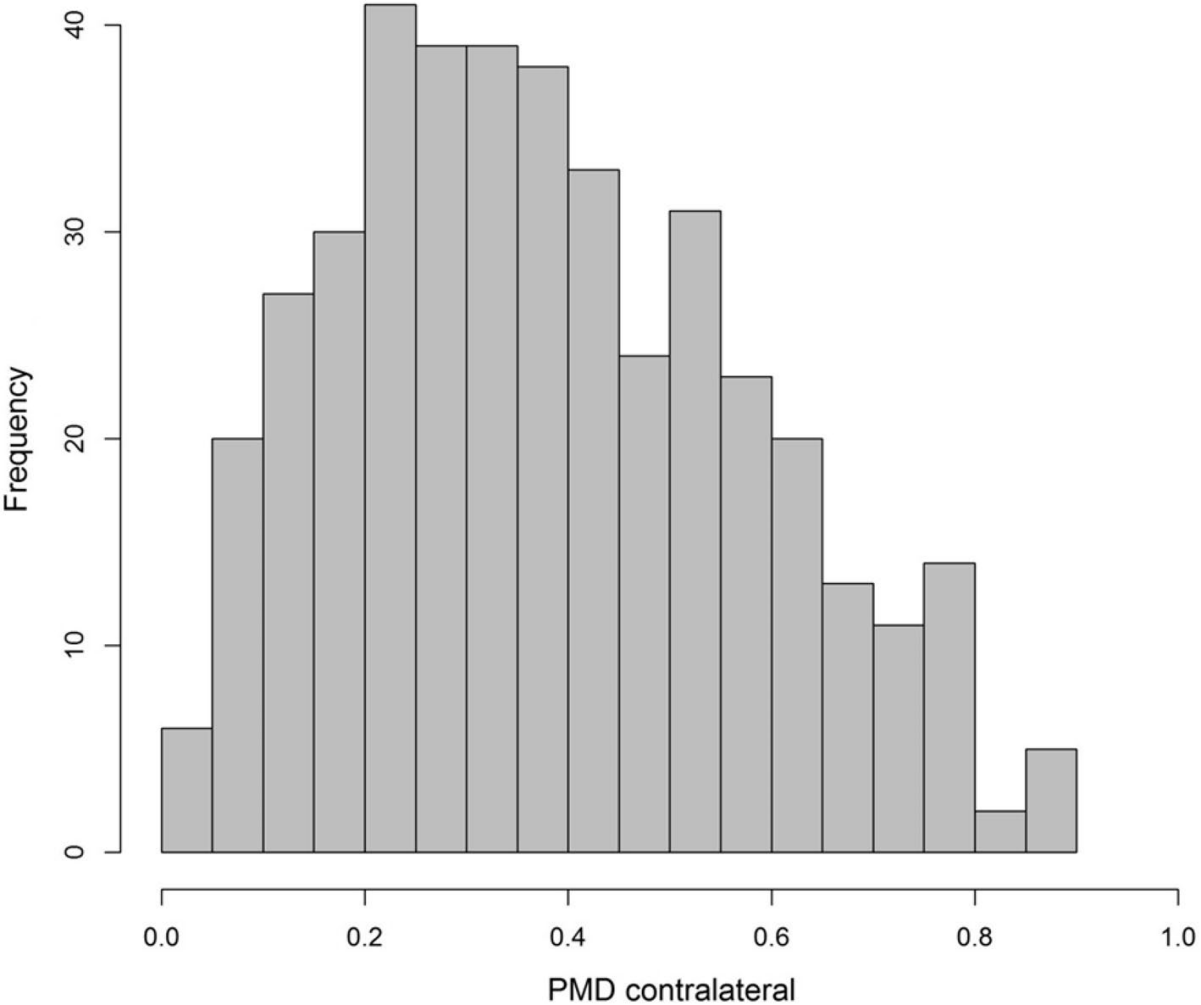
Distribution of percent mammographic density (PMD) contralateral

**Fig. 3:**
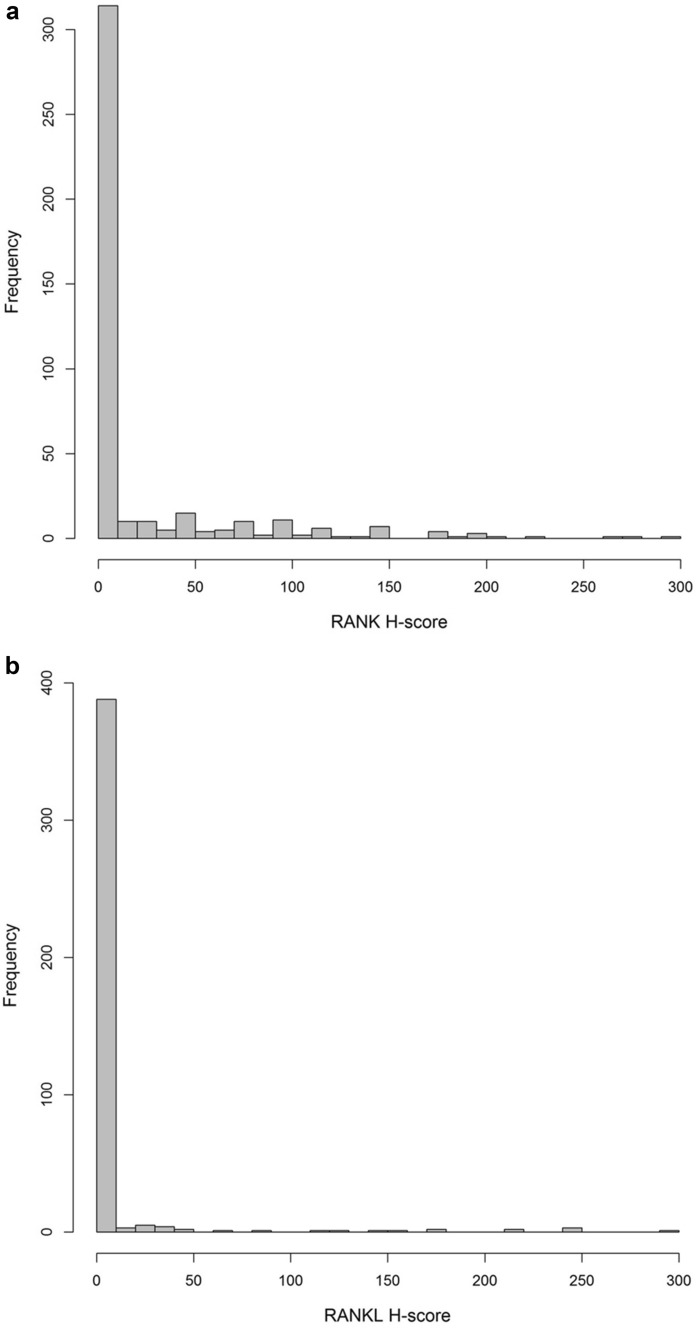
**a** Distribution of RANK H score. **b** Distribution of RANKL H score

The median PMD was 0.37 (IQR 0.24–0.53) (Table 1). The distribution of PMD is depicted in Fig. 2. In the majority of the cases, the H score for immunohistochemical assessment of RANK and RANKL was 0, while 143 patients (34.7%) showed an H score > 0 for RANK and 43 patients (10.4%) for RANKL (Table 1). The median H score of cases with a positive expression was 50 (IQR 10–100) for RANK and 30 (IQR 8–125) for RANKL. Concerning molecular subtypes, the frequency of positive RANK expression was lowest among patients with luminal A-like breast cancer (19.5%), increasing in those with luminal B-like breast cancer (26.0%), HER2-positive breast cancer (55.2%), and TNBC (67.2%). The median RANK H score among patients with detectable RANK expression increased in the same order across molecular subtypes. No subtype-specific pattern could be seen for RANKL expression (Table 2). The distribution of RANK and RANKL H score is presented in Fig. 3a, b.

**Table 2:** RANK and RANKL expression across molecular subtypes of breast cancer

Molecular subtype	RANK H score > 0				RANKL H score > 0			
	Number	Percentage	Median*	IQR*	Number	Percentage	Median*	IQR*
Luminal A-like	26/133	19.5%	15	5–50	12/133	9.0%	25	6–40
Luminal B-like	40/154	26.0%	30	10–80	20/154	13.0%	45	13.8–165
HER2-positive	32/58	55.2%	70	20–100	4/58	6.9%	15	5–81.3
Triple-negative	45/67	67.2%	80	30–150	7/67	10.4%	30	5.5–77.5
All subtypes	143/412	34.7%	50	10–100	43/412	10.4%	30	8–125

Number and percentage of patients with RANK and RANKL H score > 0 is shown across molecular subtypes of breast cancer. *Among the subset of patients with RANK and RANKL H score > 0, the median including interquartile range (IQR) is shown

**Fig. 4:**
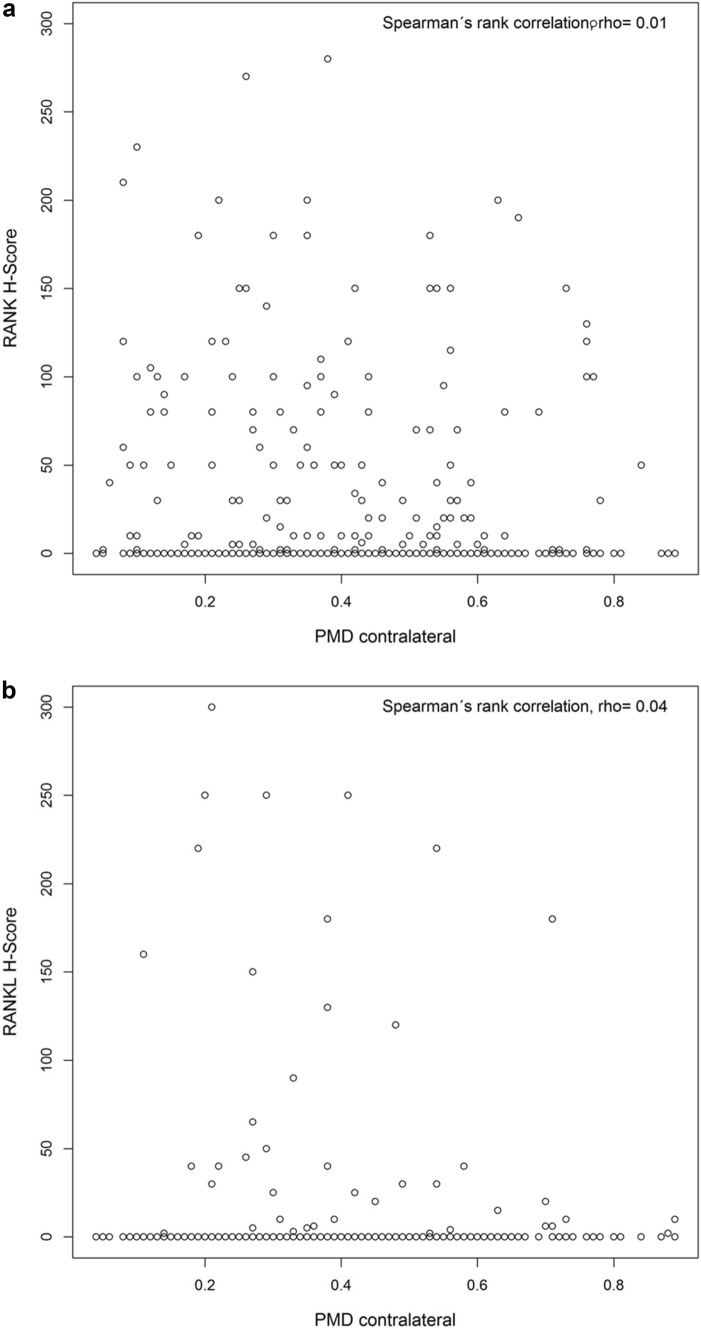
**a** Scatterplot of RANK H score and PMD contralateral. **b** Scatterplot of RANKL H score and PMD contralateral

The linear regression analysis did not show an association of PMD with RANK and RANKL expression assessed by H score (F test, *P* = 0.68). Furthermore, sensitivity analysis revealed no significant correlation between PMD and RANK (Spearman’s ρ = 0.01, *P* = 0.87) or RANKL H score (Spearman’s ρ = 0.04, *P* = 0.41). Scatterplots for PMD and RANK or RANKL H score are shown in Fig. 4a, b.

## Discussion

In this retrospectively conducted study of 412 female patients with primary breast cancer, we could not find an association of RANK and RANKL expression, as assessed by immunohistochemistry of FFPE tumor tissue samples, with PMD of the contralateral, non-diseased breast.

In a recent observational study, we linked soluble RANKL and OPG expression to breast volume changes during pregnancy in healthy women, implicating an impact on breast proliferation [33]. Likewise, different in vitro and in vivo studies revealed a progesterone- and prolactin-driven induction of the RANK/RANKL/OPG pathway, triggering the development, growth, and migration of mammary epithelial cells, and leading to tumorigenesis and metastasis [7, 9–13]. An analysis of a subcohort of prospectively observed, initially healthy, postmenopausal women of the UKCTOCS study who developed breast cancer 12–24 months after sample collection, showed that high RANKL and progesterone serum levels led to a 5.3-fold increase of breast cancer risk [34]. Few studies also confirmed an inverse relationship of OPG serum levels with breast cancer risk in cohorts of primarily premenopausal patients with a *BRCA1/2* mutation (mean age 42 years) [35, 36] as well as in the general population for primarily postmenopausal women (mean age 61 years) [37], while another investigation did not find an association in general premenopausal women (median age 44 years) [38].

Data on the association of RANK, RANKL, and OPG expression with PMD is limited. An analysis of 365 cancer-free premenopausal women described RANK serum levels to be positively correlated with PMD. The same association was found for RANKL serum levels if progesterone levels were elevated [20]. A second study of 368 postmenopausal women showed that an increase in RANK plasma gene expression led to higher volumetric percent density. Moreover, in patients with very high vs very low PMD, RANKL and surprisingly also OPG plasma gene expression were significantly upregulated, while RANKL and OPG plasma gene expression were not higher in women with heterogeneously dense breasts compared with those with almost entirely fatty breasts [18]. Another report on 43 postmenopausal confirmed higher mean PMD for those with lower serum OPG levels, and no association was identified in 57 premenopausal women [19]. In summary, the few available studies on healthy individuals propose that elevated RANK or RANKL circulating protein levels or plasma gene expression are associated with increased PMD [18, 20], while data concerning the effect of OPG expression on PMD are inconsistent [18, 19].

Data on the breast tissue expression of RANK, RANKL, and OPG are even rarer. One report demonstrated that in 48 healthy, premenopausal women, increasing RANKL gene expression in non-diseased FFPE breast tissue was associated with greater PMD [21]. In this context, it has to be noted that our study is the first one which investigated the expression of RANK and RANKL in breast cancer tissue with regard to PMD of the contralateral, healthy breast. Generally, it has been shown that tissue expression of RANK and RANKL is increased in healthy breast tissue compared with breast cancer tissue [39, 40], that tissue expression of RANKL varies with changing levels of sex hormones during the menstrual cycle [7, 41, 42], and that it is higher in premenopausal than in postmenopausal women [43, 44]. With a mean age of 58.6 years, our study collective represents primarily postmenopausal women, which could contribute to the relatively low tumor expression of RANK and RANKL.

Immunohistochemical staining of TMAs in the current study was performed with the same antibodies as has been reported in previous trials [29, 30, 45]. We detected a positive tumor expression of RANK and RANKL in 34.7% and 10.4% of the patients, respectively, and most of these had a low expression. In line with these results, a study on TMAs of 601 breast cancer patients found a positive expression of RANK in 27% and of RANKL in 6% [30], and another large analysis of TMAs of 2299 breast cancer patients from four independent cohorts (of these 777 patients with ER-negative disease) showed even lower expression of RANK and RANKL in the tumor compartment [46]. In a trial exclusively on TNBC patients, similar expression rates as in our study were identified [47]. Some other breast cancer studies reported higher expression [43, 48–50], partly with greater rates for RANK than for RANKL [30, 46–49]. The differences could be explained by varying specificity of immunohistochemical reagents or methodologies, other scoring systems, and the distribution of patient cohorts, histological subtypes, and clinical stages.

In the current study, tumor expression of RANK and RANKL was quantified as H scores. Since expression was low with 65.3% negative cases for RANK and 89.6% negative cases for RANKL, we performed a dichotomization in either negative or positive expression. The cut-off for RANK H score is different to a previously used cut-off of ≥ 8.5, which was identified as optimal for the prediction of pathological complete response and survival in a group of patients who all underwent neoadjuvant chemotherapy [30]. In contrast to this study, we investigated the association of RANK and RANKL expression with PMD in breast cancer patients of whom 55.1% received neoadjuvant or adjuvant chemotherapy and whose breast cancers had more favorable tumor characteristics.

In our study, triple-negative and HER2-positive tumors had a greater number of RANK-positives and a stronger RANK expression compared with luminal B-like and luminal A-like tumors, while no subtype-specific expression of RANKL was detected. In line with this finding, other studies correlated tumor expression of RANK predominantly with worse prognostic molecular parameters such as ER-negative, HR-negative, triple-negative, or basal-like breast cancer [30, 39, 45–49, 51], higher grading [30, 39, 46, 49, 51], and higher Ki-67 [30, 39, 46, 51]. Tumor expression of RANKL was associated with HR-positive, luminal A-like, or non-basal-like breast cancer [39, 43, 48], lower grading [39, 49], and lower Ki-67 in some studies [39, 50].

One of the strengths of our work is the inclusion of all women with incident breast cancer from clinical routine work regardless of any other criteria, reducing the risk of bias in the selection of patients and of treatment effects on PMD. Patients were recruited from a tertiary referral center in a university hospital and not from a population-based screening facility, which generally detects earlier tumor stages. The semiautomated quantification of MD, with two experienced, independent readers for all images and a mean value for PMD being used, has been validated as a robust method in different previous studies [1, 6, 24–28]. A limitation is the retrospective nature of the analysis with the potential of missing data. Several cases had to be excluded because of incomplete values in variables of interest such as PMD, RANK, and RANKL H score or due to technical issues of TMA evaluation (e.g., inadequate tumor tissue recognizable or tumor core washed off).

## Conclusion

Although a limited number of studies has described an association between RANK, RANKL, and OPG expression in serum, plasma, or healthy breast tissue with PMD, our study does not show a correlation between tumor-specific RANK and RANKL expression with PMD in patients with primary breast cancer. Since RANK/RANKL/OPG signaling appears to play a role in the development of breast cancer and since RANKL inhibition may be a novel chemoprevention strategy in women at an increased breast cancer risk, this pathway will remain under investigation of present and future trials.

## Data Availability

The datasets generated and analyzed in this study are available from the corresponding author upon reasonable request.

## Abbreviations

BMI: Body mass index
ER: Estrogen receptor
FFPE: Formalin-fixed and paraffin-embedded
HER2: Human epidermal growth factor receptor 2
HR: Hormone receptor
IQR: Interquartile range
MD: Mammographic density
OPG: Osteoprotegerin
PMD: Percent mammographic density
PR: Progesterone receptor
RANK: Receptor activator of nuclear factor kappa B
RANKL: Receptor activator of nuclear factor kappa B ligand
TMA: Tissue microarray
TNBC: Triple-negative breast cancer
